# Bis(2-cyclo­hexyl­imino­methyl-4,6-disulfanylphenolato)nickel(II) acetonitrile solvate

**DOI:** 10.1107/S1600536809032425

**Published:** 2009-08-22

**Authors:** Qiang Wang, Jian Hou, Peng Huang, Qing-Fu Zeng

**Affiliations:** a Engineering Research Center for Clean Production of Textile Dyeing and Printing, Ministry of Education, Wuhan 430073, People’s Republic of China

## Abstract

In the title compound, [Ni(C_13_H_16_NOS_2_)_2_]·CH_3_CN, the Ni^II^ atom is four-coordinated by two *N*,*O*-bidentate Schiff base ligands, resulting in a distorted tetra­hedral coordination for the metal ion.

## Related literature

For background, see: Shi *et al.* (2008 ▶); Xu *et al.* (2009 ▶). For reference structural data, see: Allen *et al.* (1987 ▶).
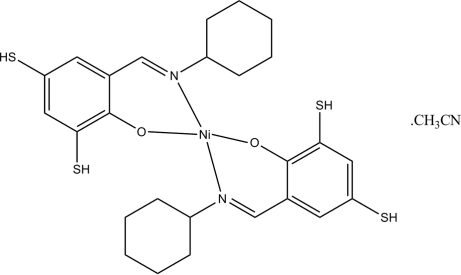


## Experimental

### 

#### Crystal data

[Ni(C_13_H_16_NOS_2_)_2_]·C_2_H_3_N
*M* *_r_* = 632.54
Monoclinic, 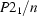

*a* = 9.483 (2) Å
*b* = 15.879 (4) Å
*c* = 20.335 (4) Å
β = 94.10 (1)°
*V* = 3054.2 (12) Å^3^
*Z* = 4
Mo *K*α radiation
μ = 0.94 mm^−1^
*T* = 296 K
0.35 × 0.27 × 0.22 mm

#### Data collection

Enraf–Nonius CAD-4 diffractometer
Absorption correction: ψ scan (North *et al.*, 1968 ▶) *T*_min_ = 0.735, *T*_max_ = 0.820
15868 measured reflections
5366 independent reflections
3750 reflections with *I* > 2σ(*I*)
*R*_int_ = 0.027
3 standard reflections every 200 reflections intensity decay: 1%

#### Refinement

*R*[*F* ^2^ > 2σ(*F* ^2^)] = 0.049
*wR*(*F* ^2^) = 0.166
*S* = 1.02
5366 reflections
348 parameters
H-atom parameters constrained
Δρ_max_ = 0.47 e Å^−3^
Δρ_min_ = −0.60 e Å^−3^

### 

Data collection: *CAD-4 Software* (Enraf–Nonius, 1989 ▶); cell refinement: *CAD-4 Software*; data reduction: *XCAD4* (Harms & Wocadlo, 1995 ▶); program(s) used to solve structure: *SHELXS97* (Sheldrick, 2008 ▶); program(s) used to refine structure: *SHELXL97* (Sheldrick, 2008 ▶); molecular graphics: *SHELXTL* (Sheldrick, 2008 ▶); software used to prepare material for publication: *SHELXTL*.

## Supplementary Material

Crystal structure: contains datablocks global, I. DOI: 10.1107/S1600536809032425/hb5049sup1.cif

Structure factors: contains datablocks I. DOI: 10.1107/S1600536809032425/hb5049Isup2.hkl

Additional supplementary materials:  crystallographic information; 3D view; checkCIF report

## Figures and Tables

**Table:** 

Ni1—O2	1.891 (3)
Ni1—O1	1.900 (3)
Ni1—N2	1.983 (3)
Ni1—N1	1.987 (3)

**Table:** 

O1—Ni1—N2	96.35 (12)
O2—Ni1—N1	95.97 (11)

**Fig. 1.**
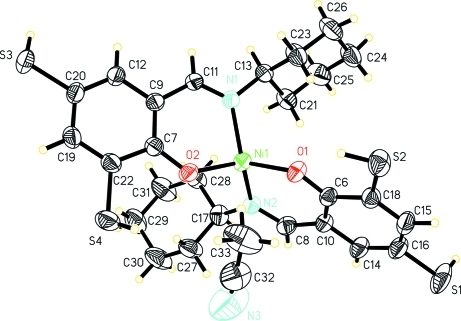
The molecular structure of (I) showing 30% probability displacement ellipsoids.

## supplementary crystallographic information

### Comment

There has been much research interest in Schiff base metal complexes due to their molecular architectures and biological activities (Shi *et al.*, 2008; Xu *et al.*, 2009). In this work, we report here the crystal structure of the title compound, (I). In (I), all bond lengths are within normal ranges (Allen *et al.*, 1987) (Fig. 1). The Ni^II^ is four-coordinated in a distort tetrahedral configuration by two N atoms and two O atoms of the Schiff base ligands.

### Experimental

A mixture of 2-hydroxy-3,5-dimercaptobenzaldehyde (372 mg, 2 mmol), cyclohexanamine (198 mg, 2 mmol) and NiCl_2_.6H_2_O (1 mmol, 236 mg) in acetonitrile (10 ml) was stirred for 1 h.  After keeping the filtrate in air for 9 d, green blocks of (I) were formed.

### Refinement

The H atoms were positioned geometrically (C—H = 0.93–0.97Å, S—H = 1.20Å) and refined as riding, with *U*_iso_(H) = 1.2*U*_eq_(carrier) or 1.5*U*_eq_(methyl C).

### Crystal data

**Table:**

[Ni(C_13_H_16_NOS_2_)_2_]·C_2_H_3_N	*F*(000) = 1328
*M**_r_* = 632.54	*D*_x_ = 1.376 Mg m^−^^3^
Monoclinic, *P*2_1_/*n*	Mo *K*α radiation, λ = 0.71073 Å
Hall symbol: -P 2yn	Cell parameters from 25 reflections
*a* = 9.483 (2) Å	θ = 9–12°
*b* = 15.879 (4) Å	µ = 0.94 mm^−^^1^
*c* = 20.335 (4) Å	*T* = 296 K
β = 94.10 (1)°	Block, green
*V* = 3054.2 (12) Å^3^	0.35 × 0.27 × 0.22 mm
*Z* = 4	

### Data collection

**Table:**

Enraf–Nonius CAD-4 diffractometer	5366 independent reflections
Radiation source: fine-focus sealed tube	3750 reflections with *I* > 2σ(*I*)
graphite	*R*_int_ = 0.027
ω/2θ scans	θ_max_ = 25.0°, θ_min_ = 1.6°
Absorption correction: ψ scan (North *et al.*, 1968)	*h* = −11→11
*T*_min_ = 0.735, *T*_max_ = 0.820	*k* = −13→18
15868 measured reflections	*l* = −24→22

### Refinement

**Table:**

Refinement on *F*^2^	Primary atom site location: structure-invariant direct methods
Least-squares matrix: full	Secondary atom site location: difference Fourier map
*R*[*F*^2^ > 2σ(*F*^2^)] = 0.049	Hydrogen site location: inferred from neighbouring sites
*wR*(*F*^2^) = 0.166	H-atom parameters constrained
*S* = 1.02	*w* = 1/[σ^2^(*F*_o_^2^) + (0.099*P*)^2^ + 1.0949*P*] where *P* = (*F*_o_^2^ + 2*F*_c_^2^)/3
5366 reflections	(Δ/σ)_max_ = 0.001
348 parameters	Δρ_max_ = 0.47 e Å^−^^3^
0 restraints	Δρ_min_ = −0.60 e Å^−^^3^

### Special details

**Table:**

Geometry. All e.s.d.'s (except the e.s.d. in the dihedral angle between two l.s. planes) are estimated using the full covariance matrix. The cell e.s.d.'s are taken into account individually in the estimation of e.s.d.'s in distances, angles and torsion angles; correlations between e.s.d.'s in cell parameters are only used when they are defined by crystal symmetry. An approximate (isotropic) treatment of cell e.s.d.'s is used for estimating e.s.d.'s involving l.s. planes.
Refinement. Refinement of *F*^2^ against ALL reflections. The weighted *R*-factor *wR* and goodness of fit *S* are based on *F*^2^, conventional *R*-factors *R* are based on *F*, with *F* set to zero for negative *F*^2^. The threshold expression of *F*^2^ > σ(*F*^2^) is used only for calculating *R*-factors(gt) *etc*. and is not relevant to the choice of reflections for refinement. *R*-factors based on *F*^2^ are statistically about twice as large as those based on *F*, and *R*- factors based on ALL data will be even larger.

### Fractional atomic coordinates and isotropic or equivalent isotropic displacement parameters (Å^2^)

**Table:**

	*x*	*y*	*z*	*U*_iso_*/*U*_eq_	
Ni1	0.71327 (5)	0.75018 (3)	0.18476 (2)	0.05582 (19)	
S2	0.26353 (13)	0.63623 (8)	0.12076 (7)	0.0863 (4)	
H2	0.3344	0.6206	0.1705	0.129*	
S3	1.23712 (14)	0.60737 (8)	0.43291 (7)	0.0908 (4)	
H3	1.3275	0.5851	0.3984	0.136*	
S4	0.79783 (18)	0.81852 (8)	0.40833 (6)	0.0977 (5)	
H4	0.6762	0.7970	0.4038	0.147*	
S1	0.13179 (17)	0.90908 (12)	−0.02558 (9)	0.1251 (6)	
H1	0.1660	0.9015	−0.0810	0.188*	
O1	0.5238 (3)	0.72316 (17)	0.15565 (13)	0.0613 (6)	
N1	0.8485 (3)	0.65868 (19)	0.16717 (14)	0.0536 (7)	
N2	0.7138 (3)	0.86444 (18)	0.14541 (15)	0.0557 (7)	
O2	0.7561 (3)	0.75360 (16)	0.27700 (13)	0.0637 (7)	
C6	0.4421 (4)	0.7675 (2)	0.11519 (18)	0.0536 (9)	
C7	0.8643 (4)	0.7192 (2)	0.30842 (18)	0.0545 (9)	
C8	0.6061 (4)	0.8918 (2)	0.10958 (19)	0.0629 (10)	
H8	0.6132	0.9463	0.0935	0.076*	
C9	0.9529 (4)	0.6585 (2)	0.28132 (17)	0.0525 (8)	
C10	0.4764 (4)	0.8483 (2)	0.09145 (18)	0.0563 (9)	
C11	0.9349 (4)	0.6301 (2)	0.21331 (18)	0.0533 (8)	
H11	0.9928	0.5860	0.2019	0.064*	
C12	1.0666 (4)	0.6232 (2)	0.32019 (19)	0.0605 (9)	
H12	1.1233	0.5827	0.3022	0.073*	
C13	0.8493 (4)	0.6203 (2)	0.10085 (18)	0.0606 (9)	
H13	0.9417	0.5941	0.0963	0.073*	
C14	0.3781 (4)	0.8914 (3)	0.0477 (2)	0.0724 (11)	
H14	0.4004	0.9444	0.0320	0.087*	
C15	0.2163 (4)	0.7773 (3)	0.0500 (2)	0.0734 (12)	
H15	0.1299	0.7534	0.0359	0.088*	
C16	0.2523 (5)	0.8559 (3)	0.0285 (2)	0.0796 (13)	
C17	0.8334 (4)	0.9231 (3)	0.1596 (2)	0.0653 (10)	
H17	0.8340	0.9642	0.1237	0.078*	
C18	0.3084 (4)	0.7349 (3)	0.0923 (2)	0.0626 (10)	
C19	1.0113 (5)	0.7079 (3)	0.4122 (2)	0.0705 (11)	
H19	1.0305	0.7247	0.4558	0.085*	
C20	1.0944 (4)	0.6480 (2)	0.3841 (2)	0.0651 (10)	
C21	0.8255 (5)	0.6871 (3)	0.04867 (19)	0.0704 (11)	
H21A	0.8994	0.7292	0.0544	0.084*	
H21B	0.7357	0.7147	0.0538	0.084*	
C22	0.9003 (5)	0.7419 (2)	0.3745 (2)	0.0652 (11)	
C23	0.7365 (6)	0.5537 (3)	0.0916 (2)	0.0792 (13)	
H23A	0.6451	0.5784	0.0983	0.095*	
H23B	0.7544	0.5096	0.1241	0.095*	
C24	0.7174 (7)	0.5820 (3)	−0.0312 (2)	0.0996 (17)	
H24A	0.7272	0.5560	−0.0738	0.119*	
H24B	0.6236	0.6063	−0.0316	0.119*	
C25	0.8253 (6)	0.6502 (3)	−0.0205 (2)	0.0934 (16)	
H25A	0.8056	0.6945	−0.0526	0.112*	
H25B	0.9182	0.6276	−0.0272	0.112*	
C27	0.8141 (5)	0.9695 (3)	0.2230 (3)	0.0917 (16)	
H27A	0.8095	0.9294	0.2588	0.110*	
H27B	0.7258	1.0005	0.2191	0.110*	
C28	0.9709 (4)	0.8773 (3)	0.1640 (3)	0.0867 (15)	
H28A	0.9835	0.8494	0.1224	0.104*	
H28B	0.9699	0.8344	0.1980	0.104*	
C26	0.7338 (7)	0.5155 (3)	0.0220 (2)	0.1025 (17)	
H26A	0.8209	0.4847	0.0175	0.123*	
H26B	0.6560	0.4759	0.0163	0.123*	
C29	1.0753 (5)	0.9852 (4)	0.2428 (3)	0.1024 (17)	
H29A	1.0789	0.9463	0.2796	0.123*	
H29B	1.1516	1.0254	0.2505	0.123*	
C30	0.9370 (6)	1.0306 (4)	0.2386 (3)	0.118 (2)	
H30A	0.9370	1.0732	0.2044	0.141*	
H30B	0.9246	1.0587	0.2801	0.141*	
C31	1.0947 (5)	0.9375 (4)	0.1802 (3)	0.113 (2)	
H31A	1.1821	0.9057	0.1849	0.135*	
H31B	1.1015	0.9770	0.1441	0.135*	
C32	0.4297 (7)	0.8572 (5)	0.2992 (4)	0.118 (2)	
C33	0.4114 (8)	0.7686 (4)	0.3059 (4)	0.136 (3)	
H33A	0.4186	0.7538	0.3518	0.204*	
H33B	0.4832	0.7396	0.2838	0.204*	
H33C	0.3199	0.7527	0.2866	0.204*	
N3	0.4466 (8)	0.9245 (5)	0.2955 (5)	0.202 (4)	

### Atomic displacement parameters (Å^2^)

**Table:**

	*U*^11^	*U*^22^	*U*^33^	*U*^12^	*U*^13^	*U*^23^
Ni1	0.0539 (3)	0.0583 (3)	0.0548 (3)	0.0049 (2)	0.0010 (2)	0.0045 (2)
S2	0.0703 (7)	0.0812 (8)	0.1054 (10)	−0.0226 (6)	−0.0069 (6)	0.0092 (7)
S3	0.0947 (9)	0.0854 (8)	0.0856 (8)	0.0120 (7)	−0.0396 (7)	0.0045 (6)
S4	0.1485 (13)	0.0862 (8)	0.0582 (7)	0.0428 (8)	0.0060 (7)	−0.0134 (6)
S1	0.0975 (10)	0.1409 (14)	0.1285 (13)	0.0185 (9)	−0.0514 (9)	0.0419 (11)
O1	0.0512 (14)	0.0626 (15)	0.0692 (17)	0.0009 (12)	−0.0019 (12)	0.0147 (13)
N1	0.0524 (17)	0.0566 (17)	0.0514 (17)	0.0010 (13)	0.0013 (14)	−0.0010 (13)
N2	0.0549 (18)	0.0538 (17)	0.0583 (18)	−0.0018 (14)	0.0027 (14)	0.0042 (14)
O2	0.0707 (17)	0.0704 (17)	0.0496 (15)	0.0234 (13)	0.0009 (13)	0.0020 (11)
C6	0.0460 (19)	0.064 (2)	0.051 (2)	0.0031 (16)	0.0047 (16)	0.0011 (16)
C7	0.065 (2)	0.0505 (19)	0.048 (2)	−0.0004 (18)	0.0033 (17)	0.0033 (16)
C8	0.071 (3)	0.056 (2)	0.061 (2)	0.0054 (19)	0.0038 (19)	0.0138 (18)
C9	0.057 (2)	0.0480 (19)	0.052 (2)	−0.0005 (16)	0.0026 (16)	0.0027 (15)
C10	0.053 (2)	0.059 (2)	0.057 (2)	0.0036 (17)	−0.0011 (16)	0.0051 (17)
C11	0.050 (2)	0.051 (2)	0.058 (2)	0.0045 (16)	0.0020 (16)	−0.0027 (16)
C12	0.059 (2)	0.057 (2)	0.063 (2)	0.0004 (18)	−0.0066 (18)	0.0019 (18)
C13	0.066 (2)	0.063 (2)	0.052 (2)	0.0069 (19)	0.0013 (17)	−0.0074 (17)
C14	0.069 (3)	0.075 (3)	0.071 (3)	0.009 (2)	−0.009 (2)	0.016 (2)
C15	0.050 (2)	0.100 (3)	0.069 (3)	0.005 (2)	−0.0070 (19)	−0.001 (2)
C16	0.069 (3)	0.094 (3)	0.073 (3)	0.022 (2)	−0.014 (2)	0.009 (2)
C17	0.065 (2)	0.061 (2)	0.070 (3)	−0.0159 (19)	0.0038 (19)	0.0089 (19)
C18	0.054 (2)	0.074 (3)	0.059 (2)	0.0018 (18)	0.0033 (18)	−0.0036 (18)
C19	0.098 (3)	0.057 (2)	0.053 (2)	−0.004 (2)	−0.015 (2)	0.0020 (19)
C20	0.069 (2)	0.057 (2)	0.067 (3)	−0.0027 (19)	−0.0175 (19)	0.0079 (19)
C21	0.088 (3)	0.067 (3)	0.057 (2)	−0.013 (2)	0.011 (2)	−0.0008 (19)
C22	0.092 (3)	0.053 (2)	0.050 (2)	0.006 (2)	0.002 (2)	−0.0014 (16)
C23	0.116 (4)	0.060 (2)	0.059 (2)	−0.018 (2)	−0.005 (2)	0.0038 (19)
C24	0.155 (5)	0.075 (3)	0.064 (3)	−0.009 (3)	−0.027 (3)	−0.005 (2)
C25	0.142 (5)	0.083 (3)	0.057 (3)	−0.002 (3)	0.019 (3)	0.004 (2)
C27	0.068 (3)	0.077 (3)	0.131 (4)	−0.008 (2)	0.017 (3)	−0.041 (3)
C28	0.060 (3)	0.097 (3)	0.105 (4)	−0.015 (2)	0.021 (2)	−0.041 (3)
C26	0.168 (5)	0.062 (3)	0.074 (3)	−0.014 (3)	−0.015 (3)	−0.010 (2)
C29	0.078 (3)	0.119 (4)	0.110 (4)	−0.035 (3)	0.008 (3)	−0.037 (3)
C30	0.101 (4)	0.096 (4)	0.158 (6)	−0.029 (3)	0.028 (4)	−0.062 (4)
C31	0.065 (3)	0.130 (5)	0.148 (5)	−0.036 (3)	0.031 (3)	−0.061 (4)
C32	0.093 (4)	0.105 (5)	0.159 (6)	−0.015 (4)	0.020 (4)	0.009 (4)
C33	0.178 (7)	0.111 (5)	0.130 (6)	−0.026 (4)	0.081 (5)	−0.016 (4)
N3	0.146 (6)	0.105 (5)	0.350 (13)	−0.024 (4)	−0.011 (6)	0.050 (6)

### Geometric parameters (Å, °)

**Table:**

Ni1—O2	1.891 (3)	C17—C27	1.507 (6)
Ni1—O1	1.900 (3)	C17—H17	0.9800
Ni1—N2	1.983 (3)	C19—C22	1.368 (6)
Ni1—N1	1.987 (3)	C19—C20	1.386 (6)
S2—C18	1.734 (4)	C19—H19	0.9300
S2—H2	1.2000	C21—C25	1.523 (6)
S3—C20	1.744 (4)	C21—H21A	0.9700
S3—H3	1.2000	C21—H21B	0.9700
S4—C22	1.731 (4)	C23—C26	1.537 (6)
S4—H4	1.2000	C23—H23A	0.9700
S1—C16	1.746 (4)	C23—H23B	0.9700
S1—H1	1.2000	C24—C25	1.495 (7)
O1—C6	1.297 (4)	C24—C26	1.513 (7)
N1—C11	1.283 (4)	C24—H24A	0.9700
N1—C13	1.481 (5)	C24—H24B	0.9700
N2—C8	1.287 (5)	C25—H25A	0.9700
N2—C17	1.480 (5)	C25—H25B	0.9700
O2—C7	1.291 (4)	C27—C30	1.532 (6)
C6—C10	1.417 (5)	C27—H27A	0.9700
C6—C18	1.417 (5)	C27—H27B	0.9700
C7—C22	1.409 (5)	C28—C31	1.531 (6)
C7—C9	1.415 (5)	C28—H28A	0.9700
C8—C10	1.436 (5)	C28—H28B	0.9700
C8—H8	0.9300	C26—H26A	0.9700
C9—C12	1.407 (5)	C26—H26B	0.9700
C9—C11	1.453 (5)	C29—C30	1.493 (8)
C10—C14	1.418 (5)	C29—C31	1.504 (7)
C11—H11	0.9300	C29—H29A	0.9700
C12—C20	1.365 (5)	C29—H29B	0.9700
C12—H12	0.9300	C30—H30A	0.9700
C13—C23	1.505 (6)	C30—H30B	0.9700
C13—C21	1.506 (6)	C31—H31A	0.9700
C13—H13	0.9800	C31—H31B	0.9700
C14—C16	1.351 (6)	C32—N3	1.085 (8)
C14—H14	0.9300	C32—C33	1.424 (9)
C15—C18	1.358 (6)	C33—H33A	0.9600
C15—C16	1.374 (7)	C33—H33B	0.9600
C15—H15	0.9300	C33—H33C	0.9600
C17—C28	1.490 (6)		
			
O2—Ni1—O1	116.53 (12)	C13—C21—H21A	109.3
O2—Ni1—N2	111.55 (12)	C25—C21—H21A	109.3
O1—Ni1—N2	96.35 (12)	C13—C21—H21B	109.3
O2—Ni1—N1	95.97 (11)	C25—C21—H21B	109.3
O1—Ni1—N1	112.76 (12)	H21A—C21—H21B	107.9
N2—Ni1—N1	125.16 (12)	C19—C22—C7	124.0 (4)
C18—S2—H2	109.5	C19—C22—S4	119.0 (3)
C20—S3—H3	109.5	C7—C22—S4	117.0 (3)
C22—S4—H4	109.5	C13—C23—C26	110.9 (4)
C16—S1—H1	109.5	C13—C23—H23A	109.4
C6—O1—Ni1	125.7 (2)	C26—C23—H23A	109.4
C11—N1—C13	118.4 (3)	C13—C23—H23B	109.4
C11—N1—Ni1	120.9 (2)	C26—C23—H23B	109.4
C13—N1—Ni1	120.7 (2)	H23A—C23—H23B	108.0
C8—N2—C17	117.7 (3)	C25—C24—C26	111.5 (4)
C8—N2—Ni1	120.8 (3)	C25—C24—H24A	109.3
C17—N2—Ni1	121.4 (2)	C26—C24—H24A	109.3
C7—O2—Ni1	125.8 (2)	C25—C24—H24B	109.3
O1—C6—C10	124.5 (3)	C26—C24—H24B	109.3
O1—C6—C18	119.4 (3)	H24A—C24—H24B	108.0
C10—C6—C18	116.1 (3)	C24—C25—C21	111.6 (4)
O2—C7—C22	119.6 (3)	C24—C25—H25A	109.3
O2—C7—C9	124.7 (3)	C21—C25—H25A	109.3
C22—C7—C9	115.8 (3)	C24—C25—H25B	109.3
N2—C8—C10	127.9 (3)	C21—C25—H25B	109.3
N2—C8—H8	116.1	H25A—C25—H25B	108.0
C10—C8—H8	116.1	C17—C27—C30	110.4 (4)
C12—C9—C7	120.3 (3)	C17—C27—H27A	109.6
C12—C9—C11	116.4 (3)	C30—C27—H27A	109.6
C7—C9—C11	123.3 (3)	C17—C27—H27B	109.6
C6—C10—C14	119.6 (3)	C30—C27—H27B	109.6
C6—C10—C8	124.2 (3)	H27A—C27—H27B	108.1
C14—C10—C8	116.2 (4)	C17—C28—C31	111.2 (4)
N1—C11—C9	127.7 (3)	C17—C28—H28A	109.4
N1—C11—H11	116.1	C31—C28—H28A	109.4
C9—C11—H11	116.1	C17—C28—H28B	109.4
C20—C12—C9	120.6 (4)	C31—C28—H28B	109.4
C20—C12—H12	119.7	H28A—C28—H28B	108.0
C9—C12—H12	119.7	C24—C26—C23	112.1 (4)
N1—C13—C23	110.7 (3)	C24—C26—H26A	109.2
N1—C13—C21	109.9 (3)	C23—C26—H26A	109.2
C23—C13—C21	109.7 (3)	C24—C26—H26B	109.2
N1—C13—H13	108.8	C23—C26—H26B	109.2
C23—C13—H13	108.8	H26A—C26—H26B	107.9
C21—C13—H13	108.8	C30—C29—C31	110.8 (5)
C16—C14—C10	120.5 (4)	C30—C29—H29A	109.5
C16—C14—H14	119.8	C31—C29—H29A	109.5
C10—C14—H14	119.8	C30—C29—H29B	109.5
C18—C15—C16	119.2 (4)	C31—C29—H29B	109.5
C18—C15—H15	120.4	H29A—C29—H29B	108.1
C16—C15—H15	120.4	C29—C30—C27	111.0 (4)
C14—C16—C15	121.4 (4)	C29—C30—H30A	109.4
C14—C16—S1	120.3 (4)	C27—C30—H30A	109.4
C15—C16—S1	118.3 (4)	C29—C30—H30B	109.4
N2—C17—C28	111.1 (3)	C27—C30—H30B	109.4
N2—C17—C27	109.7 (3)	H30A—C30—H30B	108.0
C28—C17—C27	110.3 (4)	C29—C31—C28	111.0 (4)
N2—C17—H17	108.6	C29—C31—H31A	109.4
C28—C17—H17	108.6	C28—C31—H31A	109.4
C27—C17—H17	108.6	C29—C31—H31B	109.4
C15—C18—C6	123.2 (4)	C28—C31—H31B	109.4
C15—C18—S2	119.8 (3)	H31A—C31—H31B	108.0
C6—C18—S2	117.1 (3)	N3—C32—C33	178.0 (10)
C22—C19—C20	118.4 (4)	C32—C33—H33A	109.5
C22—C19—H19	120.8	C32—C33—H33B	109.5
C20—C19—H19	120.8	H33A—C33—H33B	109.5
C12—C20—C19	120.9 (4)	C32—C33—H33C	109.5
C12—C20—S3	121.7 (3)	H33A—C33—H33C	109.5
C19—C20—S3	117.4 (3)	H33B—C33—H33C	109.5
C13—C21—C25	111.7 (4)		
